# Assessing preoperative hope and expectations related to functional neurosurgery: a new questionnaire

**DOI:** 10.1186/s40359-022-00766-z

**Published:** 2022-03-04

**Authors:** Michalina Radomska, João Flores Alves dos Santos, Kerstin Weber, Marc Baertschi, Pierre R. Burkhard, François Herrmann, Sanaâ Belayachi, Nicolas Favez, Alessandra Canuto

**Affiliations:** 1 University of Geneva, Geneva, Switzerland; 2 Geneva University Hospitals, Geneva, Switzerland; 3 University of Lausanne, Lausanne, Switzerland; 4 University of Liège, Liège, Belgium

**Keywords:** Epilepsy surgery, Deep brain stimulation, Preoperative expectations, Hope, Questionnaire

## Abstract

**Background:**

Despite successful functional neurosurgery, patients suffering from epilepsy or Parkinson’s disease may experience postoperative psychological distress and social maladjustments. Difficulties in coping with postoperative changes, even positive ones, have shown to be related to patients’ presurgery cognitive representations (i.e., expectations, hope). The aim of this study was to develop an instrument assessing various key features of surgery outcomes’ representations, namely the Preoperative Hope and Expectations Questionnaire (PHEQ).

**Methods:**

Participants were patients (*n* = 50) diagnosed with Parkinson’s disease (*n* = 25) or epilepsy (*n* = 25), candidates for functional neurosurgery (i.e., Deep brain stimulation, anterior temporal lobectomy). Two to three weeks before the planned surgery, they were administrated items assessing their actual state, preoperative expectations, and hope regarding surgery outcomes. They also completed measures assessing optimism, quality of life and mood.

**Results:**

Exploratory analysis resulted in a 14-item version of the PHEQ composed of two factors (*abstract representations*, including psychological well-being and *concrete representations*, such as direct surgery outcomes). The PHEQ demonstrated high internal consistency and good convergent validity. Patients were more prone to express postoperative improvements in terms of hope rather than expectations. They generally focused on concrete rather than abstract features, although patients with Parkinson’s disease had higher abstract future-oriented representations.

**Conclusions:**

The PHEQ presents satisfactory psychometric properties and may be considered as a reliable instrument for research and clinical practice.

**Supplementary Information:**

The online version contains supplementary material available at 10.1186/s40359-022-00766-z.

## Background

Bilateral subthalamic nuclei deep brain stimulation (DBS) is known to reduce motor symptoms as well as dopaminergic-related complications in advanced Parkinson's disease (PD) [1]. While successful functional neurosurgery leading to the sudden alleviation of symptoms is expected to significantly improve patients’ quality of life (QoL), growing evidence suggest that such positive effect is questionable [2–5]. This phenomenon has been well documented in surgical treatment of medically intractable epilepsy. More specifically, despite successful anterior temporal lobectomy (ATL) and alleviation of seizures, some patients experience postoperative psychological and socio-professional maladjustments (e.g., difficulties discarding sick role behaviors,^1^ family dysfunctions, occupational disabilities), leading to major deterioration in their postoperative QoL [6, 7]. Future-oriented cognitions, such as hope and expectations regarding surgery outcomes, has been suggested to play a key role in postoperative psychosocial adjustment process.

Patients’ expectations can be broadly defined as future-directed beliefs about the occurrence of a specific outcome. Treatment expectations have been shown to play a role in different kind of medical procedures such as cardiac surgery [8, 9], total knee arthroplasty [10–12], or shoulder surgery [13, 14]. Such preoperative representations have been significantly related to the success of rehabilitation [8, 15], to the level of postoperative functional recovery [16–18] and to postoperative QoL [19–21].

In a similar vein, several studies have suggested that expectations might play a key role in placebo and nocebo effects, with positive expectations being linked to a variety of improved health outcomes in PD [22–25] or in epilepsy treatment [26]. While this suggests that optimistic expectations might have positive effects on patients’ perception of treatment outcomes, some authors have voiced concern regarding expectations that are too optimistic, which may lead to worsening patients’ perception of treatment outcome [27]. Furthermore, it should be noted that placebo and nocebo effects in PD related symptoms appear to be applicable only for a subgroup of patients and for specific symptoms [28, 29].

Additionally, the specificity of preoperative goals may play an important role in postoperative QoL and well-being. According to goal-related theories [30], goals may vary according to their degrees of abstraction, ranging from concrete (e.g., functional aspects of everyday life) to abstract goals (i.e., goals related to self-representations and interpersonal satisfaction). Abstract goals are known to be more difficult to achieve, thereby leading to repeated goal failures [31]. Indeed, endorsing goals at a high level of abstraction has been related to an increased propensity to experience psychological distress, as compared to endorsing goals at concrete levels. Consistently, candidates for functional neurosurgery with excessively high or unspecific treatment expectations (e.g., being normal, feeling like myself again) have been reported to more frequently experience postoperative psychological distress and a general dissatisfaction with surgery outcomes [5, 32–34].

Numerous studies have explored preoperative expectations of candidates for DBS or ATL (see Table 1). Nevertheless, these studies vary widely in conceptual and methodological approaches, ranging from qualitative design with structured or semi-structured interviews [33, 35–37] to *ad hoc* questionnaires [38–40], and only a few studies have used validated instruments [12, 41, 42]. Some studies have provided a modified satisfaction scale or modified standard measures of symptoms used as an expectation scale [43, 44], in which patients are asked to rate for each question the current symptom severity (e.g., ranging from *no problem* to *severe problem*) and the expectation for change after treatment (e.g., ranging from *expected to be very much worse* to *expected to be very much improved*). However, the transferability of dimensions from satisfaction or functional state to the measurement of expectations has received limited justification.

**Table 1:** Characteristics of reviewed studies exploring expectations of candidates for functional neurosurgery (DBS and ATL)

References	Surgery	Sample	Method	Domain of assessed preoperative representations
Reddy et al. [40]	DBS	22 patients with PD	Ad hoc questionnaire: Patient Reported Outcomes in Advanced Parkinson’s disease scale (PRO-APD) Patients were asked to rate for each question: (1) the symptom severity, (2) the expectation for change after therapy: − 3 (expected to be very much worse), to +3 (expected to be very much improved).	Motor domain: tremor, stiffness, off periods, dyskinesia, freezing, dystonia, speech, balance Non-motor domain: swallowing, sleep, bowels, bladder, pain, fatigue, sexual function Cognitive/psychological domain: concentration, memory, impulsive behavior, hallucinations/psychosis, mood, anxiety, apathy Social and ADL: self-care, work, leisure/hobbies, socializing
Maier et al. [33]	DBS	30 patients with PD	Semi-structured interview regarding preoperative expectations	Health: motor improvement, reduction of medication, improvement of walking, improvement of tremor, less dyskinesia, improvement of general health ADL: carry out hobbies, car driving, trips, travels, Social: more socializing, improvement of partnership Psychological: improvement of quality of life, improvement of mental state
Nisenzon et al. [43]	DBS	148 patients with PD	Modified version of the Patient-Centered Outcomes Questionnaire (PCOQ-PD), patients were asked to rate for each domain: (1) Usual levels of difficulty over the past week, (2) success criteria, (3) expectations, (4) importance	Health: Pain, fatigue, tremor, stiffness in limbs, slowness in movement, walking problems, sleep Psychological: Emotional distress, thinking ADL: Interference with daily activities (work, leisure)
Törnqvist et al. [37]	DBS	8 patients with essential tremor 8 patients with PD	Semi-structured interview Standardized open questions: What motor/social activities can you perform today/ would you like to be able to perform when your tremor has decreased?	Motor activity: housekeeping, hygiene, eating and drinking, writing, working, leisure activities Social activity: being with other people, participating in social activities
Baca et al. [39]	ATL	389 patients with epilepsy	Ad hoc questionnaire based on the literature and clinical experience 12 items, each item rated on a scale from 1 (not at all important) to 10 (extremely important)	ADL: driving limitations, limitations in bicycling, swimming, other physical activities Social: participation in social situations Health: level of fatigue, cosmetic physical aspects, pregnancy concerns, having to take epilepsy medications Psychological: emotional well-being, memory problems, language problems, concentration or attention problems, economic worries
Baca et al. [39]	ATL	391 patients with epilepsy	Interview Open-ended questions about expectations for surgical outcome - “In what ways do you feel limited by your epilepsy?” - “What do you most hope to change as a result of this surgery?”	Expectations endorsed by > 15% of the sample: driving, job/school, independence, seizure cessation, social functioning, quality of life, medication discontinuance, physical activities, cognition Expectations endorsed by less than 15% of the sample: embarrassment/stigma, emotional, fatigue, general health, family planning, no limitations
Salgado et al. [42]	ATL	73 patients with epilepsy before surgery 63 patients with epilepsy after surgery	Validation of the pre-surgery expectations questionnaire 18 yes/no questions	Health: take less anti-epileptic medication, be healthy ADL: drive, work or study, take care of my house / of my family, have fun, be safe to hang out alone Social: have children, improve my social life, marry, improve my sexual life, be accepted by my family Psychological: improve my memory, be happy, be less worried, feel free, be less nervous, feel ordinary
Wheelock [48]	ATL	32 patients with epilepsy 17 significant others	Semi-structured Interview about Epilepsy Surgery (SIAES) (1) Ways in which seizure elimination would affect the patient’s relationships with significant others (2) …would be a good or positive change (3) …would be a difficult or negative change	Have more friends, be less dependent, others will worry less, marital and family relations will improve Be able to drive, to work, continue education, do more activities, mood improvement, risk of injury or accident eliminated, reduces medication, anxiety eliminated, not feel as seek, not feel tired Negative side effects of surgery, less attentions of others, face new responsibilities, no longer need of significant other
Wilson et al. [36]	ATL	60 patients with epilepsy	Standardized, semi-structured clinical interview (1) What is the main reason you have sought surgical intervention? (2) Do you see the operation as a chance to change your life? (3) Have you made any postoperative plans? (4) Do you plan on engaging in any new activities/ hobbies postoperatively?	Health: seizure ablation, medication ADL: driving, employment, independence, new activities Psychological: self change, general improvement Social: family, relationships
Rose et al. [38]	ATL	17 patients with epilepsy	Ad hoc questionnaire The Epilepsy Expectations Questionnaire (EEQ) Responses are based on future expectations (1 year), rated on a 7-point Likert-type scale ranging from 1 (I do not expect this) to 7 (I very strongly expect this)	Physical health, epilepsy medication, seizure frequency Mood, quality of life Social adjustment Driving, occupation

*PD* Parkinson’s disease, *DBS* deep brain stimulation, *ATL* anterior temporal lobectomy, *ADL* activities of daily living

Finally, most studies have failed to make a distinction between hope and expectation, while they are related yet distinct constructs [45]. Patients’ expectations are often conceptualized to be situational (i.e. treatment specific), in contrast to hope and optimism which are described as dispositional [46]. However, in medical situations like elective surgery, patients evaluate their hope and optimism in a situational way, referring to their upcoming treatment [47]. Furthermore, Haanstra et al. [47] tested the possibility that there is a strong general “outlook on future” factor that underlies measures of treatment credibility, treatment expectancy, optimism, and pessimism, that each account for unique variance above this general factor. Their results showed that this model fit the data better than any other model tested and that there is a strong general factor that accounts for a large amount of the variance.

However, Uhlmann et al. [49] highlighted an important distinction between expectation (probabilistic beliefs that something will happen) and hope (desire that the specific outcome would occur). More specifically, the author suggested that patients' expectations and hope pertain to two distinct perceptual dimensions: expectancy and value. Expectancy primarily reflects a perception that the occurrence of a given outcome is likely. Patients’ hope, in contrast to expectations, primarily reflect a valuation, a perception that a given outcome is desired. An outcome may be wanted but not expected (e.g., *I hope my disease will be cured, but I do not expect that*) or, inversely, expected but not desired (e.g., *I expect to receive, but do not want, a painful injection*). More recent studies further suggested to differentiate probability expectations (rational projections) and idealized expectations (or hopes) in exploring patients’ expectations in clinical trials [50]. In their study based on cognitive interviews, patients defined hope as what they wished for or wanted to occur at the highest levels of aspiration, unconstrained by reality, prior knowledge or experience, and expectations as the most realistic projections of what might happen based on prior experience and illness history. This distinction was consistent across participants.

To sum up, patients’ future-oriented cognition constitutes an important determinant of clinical outcomes following functional neurosurgery. Discrepancies between anticipated outcome and postsurgical reality, even in the case of significant symptoms reduction, may yield to disappointment and psychosocial maladjustments [51]. Although several tools have been proposed to explore preoperative representations of candidates for DBS or ATL, the nature of such representations (expectation vs. hope) and their content (concrete vs. abstract) failed to be assessed properly. The aim of the present study was to develop an instrument assessing the various key features of prior representations related to surgery outcomes, namely the Preoperative Hope and Expectation Questionnaire (PHEQ). Finally, this study aimed to explore whether preoperative future-oriented representations vary according to the type of functional neurosurgery (DBS vs. ATL).

## Materials and methods

### Participants and procedure

Patients diagnosed with PD or epilepsy and potential candidates for functional neurosurgery were recruited from the University Hospitals of Geneva in Switzerland. Inclusion criteria were a DBS or epilepsy surgery medical indication established by neurologist, neurosurgeon, psychiatrist and neuropsychologist. The main selection criteria for DBS surgery were disabling motor complications of dopaminergic treatment, the absence of dementia (based on a cutoff score of 130 on the Mattis Dementia Rating Scale), and severe depression with suicidal ideations. Motor symptoms were assessed before surgery using the Unified Parkinson’s Disease Rating Scale III (UPDRS III, [52]). The selection for ATL was a thorough procedure aimed at identifying potential candidates for surgery by determining the risk-benefit ratio for each patient. Patients clinically accepted for DBS or epilepsy surgery were invited to participate in the present study. They were selected from the French speaking community since self-administered questionnaires are in French. Based on these criteria, 50 patients (32 males and 18 females) aged between 18 and 73 (Mean of overall sample: 46.16 years, *SD* = 17.05) were selected for the present study. Twenty-five patients with PD (17 men and 8 women; mean age: 59.60 years, *SD* = 7.41) were candidates for DBS, and 25 patients with epilepsy (15 men and 10 women; mean age: 32.72 years, *SD* = 12.75) were candidates for ATL.

Informed consent was obtained from all participants following a full explanation of the experimental procedure. Detailed written and oral instructions explained that participants would be asked questions about different aspects of their everyday life as well as regarding their programmed neurosurgery. They were participating on a voluntary basis. 2–3 weeks before the planned intervention, participants completed the PHEQ and all the measures described below. The order of questionnaires presentation was randomized to counterbalance order effects.

### Instruments

#### The Preoperative Hope and Expectation Questionnaire (PHEQ)

The process by which the PHEQ has been developed is described in the present section. Psychometric properties of the PHEQ (factorial structure, internal consistency, and convergent validity) are reported in the Results section (see Sect. 3). The external validity of the final version of the PHEQ was assessed by examining its relationships with measures of optimism, mood, mental and physical QoL. A high level of hope and expectations was expected to be correlated to dispositional optimism [47] and negatively correlated to anxio-depressive symptoms [53]. Additionally, concrete hope and expectations were expected to be specifically connected to physical QoL, while abstract hope and expectations to mental QoL.

*Item selection.* A qualitative review of studies exploring preoperative expectations on DBS and ATL populations by means of questionnaires, interviews and semi-structured interviews was conducted in order to identify and characterize items aimed at exploring preoperative expectations. For that purpose, items of selected tools (see Table 1) were classified according to the supra-ordered semantic category (e.g., mobility, personal care, hobbies, self, etc.). This analysis suggested that preoperative expectations focused mainly on four distinct life domains: (1) physical and mental state; (2) autonomy in daily living activities; (3) psychological and emotional well-being; and (4) social-relational life. Based on the identification of the various life domains content, an initial pool of 24 items has been generated.

All items consisted in affirmations regarding the above-mentioned life domains. Any disease-specific reference (e.g., tremor, stiffness, dyskinesia, freezing, dystonia, fatigue, seizures, etc.) has been systematically replaced by the general term of *reduction of symptoms*. It is worth noticing that expectation and hope, which are in fact two distinct concepts [49], appeared to be mixed up in previous measures. Thus, in order to examine expectation and hope separately, each item has been framed in the context of realistic expectations (e.g., *Regarding physical pain, I realistically expect…*) and in the context of hope/desire (e.g., *Regarding physical pain, I really hope for…*), and rated on a 5-point scale (0 = *no improvement at all* to 4 = *total improvement or symptom relief*). Additionally, each item has been assessed regarding actual state (e.g., *I have physical pain*), by means of a 5-point scale (0 = *not at all* to 4 = *extremely*).

*Qualitative evaluation of the initial pool of item.* Three judges (a neurologist, a psychiatrist and a neuropsychologist), who were familiar with the concept of preoperative expectations, were asked to rate the level of clarity and consistency of each item. Based on the judges’ evaluation, 6 items were discarded as they appeared irrelevant (*pregnancy concerns, others’ worries, new activities, economic worries, general health improvement, risk of injury*), 4 items were replaced by 2 more general items (the item *To be able to participate in leisure activities* included *sports, travel, etc.*; the item *To be able to work*, included *professional activity, housework, etc.*). Additionally, 4 new items were generated based on experts’ proposals in order to explore more precisely issues frequently reported by patients in clinical settings (*physical appearance, ability to enjoy life, feeling comfortable in social situations, achieve projects*). The new 20-item form was then administered to 10 candidates for DBS (*n* = 5) and ATL (*n* = 5). A free response section was included at the end of the questionnaire allowing respondents to write down any additional expectation that did not appear in the PHEQ. Based on patients’ responses, two new items were added (*To feel more like myself* and *To be like everyone else*).

*The PHEQ.* Based on experts’ and patients’ evaluation of the initial item pool, a preliminary version of the PHEQ consisted in 22 items assessing several life domains (see Table 3 for an overview of items content) varying in the level of abstraction. The PHEQ is composed of three parts allowing to explore (a) patients’ current state (Actual State, AS), (b) patients’ realistic prediction of outcomes (Preoperative Expectations, PE), and (c) patients’ wishes or desires concerning surgery outcomes (Preoperative Hope, PH). The first PHEQ dimension (AS) started with the following instruction: “*This part of the questionnaire assesses your actual state regarding different aspects of your life, considering the presence of your disease. Please answer all questions based on the past 4 weeks”.* The participants were instructed to assess their actual state for the 22 items content (e.g., *I have physical pain*), by means of a 5-point scale (0 = not at all to 4 = extremely); six items are reverse-scored (i.e., items 2, 12, 19, 20, 21 and 22). The second PHEQ dimension (PE) consisted in the 22 items content framed in the context of realistic expectations (e.g., *Regarding physical pain, I realistically expect*…) preceded by the following instruction: “*This part of the questionnaire assesses your expectations regarding the results of the intervention. What interests us is not the ideal outcome you would like to achieve, but the change that you realistically expect or believe most likely, based on different information you may have obtained”*. All items were rated by means of a 5-point scale (0 = no improvement at all to 4 = total improvement or symptom relief). Finally, the third PHEQ component (PH) consisted in the 22 items content framed in the context of hope/desire (e.g., *Regarding physical pain, I really hope for*…), and rated on a 5-point scale (0 = no improvement at all to 4 = total improvement or symptom relief). This PHEQ dimension was introduced with the following instruction: *“This part of the questionnaire assesses your hope regarding the results of the intervention. What interests us is not the realistic outcome you would expect, but the change that you really hope to achieve following the surgery”*. The AS dimension of the PHEQ was presented first. Then, half of the participants completed the PE before the PH dimension, and the other half completed the PH before the PE dimension.

#### Other measures

*Quality of life.* The French version of the Medical Outcome Study Short Form (MOS-SF-36, [54]) was administered in order to assess patients’ subjective QoL. This self-report measure consists of 36 questions about QoL and care outcomes. It evaluates eight dimensions, including the Physical Component Summary score (PCS) and the Mental Component Summary score (MCS). Each subscale’s scores range from 0 (*worst condition*) to 100 (*best condition*). In the present study, Cronbach’s alphas indicate excellent internal consistency for the PCS (.94) and the MCS (.91) measures.

*Dispositional optimism.* The French version of the Life Orientation Test Revised (LOT, [55]) was administered in order to assess dispositional optimism. This scale consisted of 10 items, rated on a 5-point scale (0 = *strongly agree* to 4 = *strongly disagree*), assessing the persons’ expectations regarding the favorability of future outcomes (e.g., *In uncertain times, I usually expect the best*). The dispositional optimism is a personality characteristic relatively stable across time. In the present study, Cronbach’s alpha indicates acceptable internal consistency for the LOT-Optimism measure (.78).

*Mood.* The French version of the Hospital Anxiety and Depression Scale (HADS, [56]) was administrated in order to examine participant’s mood status. The HADS is composed of 14 items measuring anxiety and depression symptoms. Participants had to determine to what extent the situation described in each particular statement applied to them during the last 7 days, using a 4-point scale (0 = *not at all*; 3 = *extremely*). Seven items assess the respondents’ state of depression (HADS-D), while the 7 remaining items constitute a self-reported measure of general anxiety (HADS-A). In the present study, Cronbach’s alphas indicate good to acceptable internal consistency for the HADS-A (.85) and HADS-D (.78) measures.

### Statistical analyses

Two exploratory factor analyses were performed to select items according to their level of abstraction (concrete vs. abstract), on PH and PE measures separately, since items content are identical across the two measures. The principal component analysis (PCA) method was used to extract factors from the correlation matrix of each PHEQ measure. The extraction method is preferred as a method for data reduction, since initial variables are transformed into the smaller set of linear combination. The Kaiser-Meyer-Olkin (KMO) method was used to measure sampling adequacy, and Bartlett’s test of sphericity was computed to test the null hypothesis that the variables in the correlation matrix are uncorrelated. A KMO between .50 and 1.0 and a significant Bartlett's test of sphericity are considered appropriate for factor analysis [57]. Considering the small size of the sample, factor analyses have been conducted by means of Bayesian estimations [58], using the JASP software. The reliability of each PHEQ measure was then examined with Cronbach’s alpha. Convergent validity has been explored by means of Pearson’s correlations and regression analyses. Finally, future oriented cognitions were explored across the two groups of patients by means of a factorial ANOVA.

## Results

Descriptive statistics for the entire sample and for each group of patients on all the variables of interest are reported in Table 2. The two groups of patients differed on age (*t*_48_=-9.12, *p*<.001), physical QoL (*t*_43_=-6.73, *p*<.001) and disease duration (*t*_43_=3.41, *p*<.001). There was no difference in mental QoL, in symptoms of anxiety and depression, in level of education and in optimism.

**Table 2:** Demographic and clinical characteristics of Patients in the entire sample and in each group (epilepsy and Parkinson’s Disease (PD))

Dependent variables	Groups of patients		
	Whole sample (*n*=50)	Epilepsy (*n*=25)	PD (*n*=25)
Age	46.16 (17.05)	32.72 (12.75)	59.60 (7.41)
Level of education	12.57 (4.26)	12.00 (2.83)	13.33 (5.65)
Disease duration	14.00 (7.78)	17.08 (8.88)	10.78 (4.79)
AS-total score	34.00 (7.67)	37.56 (6.95)	30.44 (6.76)
PE-total score	21.00 (11.38)	17.12 (8.53)	24.88 (12.66)
PH-total score	28.04 (13.00)	23.92 (10.39)	32.16 (14.20)
HADS-D	5.47 (3.24)	4.83 (3.26)	6.13 (3.15)
HADS-A	7.75 (4.01)	7.91 (4.18)	7.59 (3.91)
MOS-SF-PCS	43.89 (10.95)	51.48 (7.68)	35.95 (7.78)
MOS-SF-MCS	40.20 (9.69)	40.69 (10.39)	39.95 (9.11)
LOT-optimism	16.66 (4.31)	16.68 (4.59)	16.64 (4.11)

*PE* Preoperative expectations, *PH* Preoperative Hope, *HADS-A* Hospital Anxiety and Depression Scale - Anxiety, *HADS-D* Hospital Anxiety and Depression Scale - Depression, *MOS-SF-PCS* Medical Outcome Study - Short Form - Physical Component Summary, *MOS-SF-MCS* Medical Outcome Study - Short Form - Mental Component Summary, *LOT* Life Orientation Test

### Factor structure

The item-total correlations for the 22 items ranged from − .06 to .73, with a mean of .28 for the preliminary PE, and from .09 to .74 with a mean of .27 for the preliminary PH. Univariate normality was explored for the 22 items of preliminary PE and PH measures by calculating the skewness and kurtosis of each item for each measure. The results showed that skewness ranged from −.70 to 1.86 for preliminary PE and from − 1.78 to 1.25 for preliminary PH; while kurtosis ranged from − 1.62 to 2.91 for preliminary PE and from − 1.62 to 2.78 for preliminary PH, indicating no strong deviation from normality (absolute values are considered to be extreme for skewness greater than 3 and kurtosis greater than 20; [59]).

In order to classify items according to their level of abstraction (i.e., concrete vs. abstract), a factor analysis was conducted on each PE and PH preliminary measures. The KMO measure of sampling adequacy and Bartlett's test of sphericity indicated that the 22 items of the preliminary PE measure (KMO = .73, Bartlett's χ^2^ = 613.37, *p* < .0001) and the 22 items of the preliminary PH measure (KMO = .74, Bartlett's χ^2^ = 543.06, *p* < .0001) were adequate for factor analysis. The PCA method was used for extracting the factors from the correlation matrix of each PHEQ measure. For both the PE and the PH measure, two components were extracted, by means of a promax rotation. PCA conducted on PE measure yielded to the identification of one main component explaining 64.9% of the total variance and a secondary factor accounting for 33.1% of the total variance. PCA conducted on PH measure also yielded to the identification of one main component explaining 66.9% of the total variance and a secondary factor accounting for 26.4% of the total variance. In order to compare more directly scores on the PH and on the PE measures, and based on a factor loading cut off of .40, only items loading consistently across the two PHEQ dimensions were retained. Thus, factor 1 included items 3, 4, 7, 9, 10, 11, 12, 17, 18 and 21, and factor 2 encompassed items 1, 2, 13, 22 (see Table 3). Items loading on factor 1 were related to self-independence, self-representations, and social/relational life, rather than direct surgery outcomes; consequently, Factor 1 was labeled “Abstract”. Whereas items loading on factor 2 were more directly connected to surgery outcomes (i.e., To be satisfied with my life, To reduce symptoms of my disease, To be satisfied with my intellectual functioning, To get off medications); Factor 2 was thus labeled “Concrete”. Item characteristics for the two future oriented PHEQ dimensions are presented in the Additional file 1: Table A1.

**Table 3:** Factor loadings for the 22 items

#	Item	PE		PH	
		RC1	RC2	RC1	RC2
1	To be satisfied with my life	− 0.028	**0.662**	0.088	**0.742**
2	To reduce symptoms of my disease	− 0.406	**0.430**	− 0.142	**0.671**
3	To be independent in my personal care	**0.734**	− 0.050	**0.750**	− 0.301
4	To feel good about myself	**0.644**	− 0.051	**0.603**	0.136
5	To be satisfied with my relationship / romantic life	0.378	0.413	0.657	0.083
6	To be able to travel alone (e.g., driving, taking public transport)	0.317	0.073	0.235	0.039
7	To be satisfied with my physical appearance	**0.900**	− 0.173	**0.755**	− 0.359
8	To get better sleep quality	0.160	0.349	0.311	− 0.098
9	To be satisfied with my social life (family, friends)	**0.740**	0.065	**0.726**	− 0.043
10	To be able to achieve my projects	**0.588**	− 0.132	**0.438**	0.386
11	To be able to participate in leisure activities (e.g., sports, travel)	**0.594**	0.221	**0.588**	0.030
12	To feel more like myself	**0.909**	− 0.299	**0.734**	0.012
13	To be satisfied with my intellectual functioning (e.g., concentration, memory)	− 0.075	**0.848**	0.169	**0.640**
14	To be satisfied with my sex life	0.296	0.380	0.660	0.002
15	To be able to work (professional activity, housework)	0.368	0.430	0.558	0.106
16	To be like everyone else	0.661	0.022	0.366	0.191
17	Not to experience negative feelings (e.g., sad, anxious)	**0.733**	− 0.113	**0.660**	0.078
18	To feel comfortable in social situations (e.g., outings, parties)	**0.643**	0.218	**0.703**	0.097
19	To be able to enjoy life	0.361	0.442	0.646	0.327
20	To be less tired, have more energy	0.344	0.321	0.593	− 0.106
21	To reduce physical pain	**0.411**	0.248	**0.557**	− 0.087
22	To get off medications	− 0.536	**0.909**	0.255	**0.797**

Bold values indicate items loading consistently across the two PHEQ dimensions

### Reliability and construct validity

Cronbach’s alphas indicated good internal consistency for all the PHEQ measures (AS-Total score: .81; PE-Total score: .85; PH-Total score: .84). Pearson’s correlations were first computed in order to examine inter-correlations between the PE-Total score, the PH-Total score and AS measure. These analyses revealed that the measures of expectations and hope are highly correlated with each other (*r* = .81, *p* < .001; 95% CI: 0.69, 0.89), consistent with the idea that they are linked constructs. AS-Total score was negatively related to both expectations (*r* = − .46, *p* > .001; 95% CI: − 0.65, − 0.21) and hope (*r* = − .60, *p* < .001; 95% CI: − 0.75, − 0.38), supporting the idea that dissatisfaction regarding AS may lead to increased expectations and desire of substantial changes following neurosurgery. Pearson’s correlation analyses also revealed that age was moderately related to both expectations (*r* = .38, *p* = .007; 95% CI: 0.11, 0.59) and hope (*r* = .38, *p* = .005; 95% CI: 0.12, 0.60). The disease duration was negatively related to the levels of preoperative hopes and expectations (respectively r = − .34, *p* = .019; 95% CI: − 0.57, − 0.06 and r = − .35, *p* = .014; 955% CI: − 0.58, − 0.07). There was no relationship between the PHEQ measures and the level of education (*p*s >.265). There was no gender effect on PHEQ measures (*p*s >.136).

Finally, Pearson’s correlations computed to examine convergent validity revealed that generalized optimism was related to both expectations (*r* = .40, *p* = .004; 955% CI: 0.14, 0.61) and hope (*r* = .49, *p* < .001; 955% CI: 0.25, 0.68), which is consistent with previous studies [45]. There was no correlation between depression and anxiety dimensions of the HADS and the PHEQ measures (*r*s < .22, *p*s > .63). Finally, the physical QoL dimension (PCS) of the MOS-SF was negatively correlated to both expectations (*r* = − .59, *p* < .001; 955% CI: − 0.76, − 0.36) and hope (*r* = − .46, *p* < .001; 955% CI: − 0.68, − 0.20) measures. The mental QoL (MCS) was negatively associated with PH (*r* = − .41, *p* = .005; 955% CI: − 0.63, − 0.13) but not with PE (*r* = − .17, *p* = .265; 955% CI: − 0.44, 0.13).

Considering the potentially confounding influences of the intercorrelations between all the variables of interest, zero-order correlations cannot determine the independent contribution of each measure (i.e. once the effect of the other variables has been removed). Hence, to investigate the specific relationship between PHEQ measures (PE-Total score, PH-Total score) and the other variables of interest (age, AS assessment, HADS mood measures, mental and physical QoL and optimism), two regression analyses were performed. The PHEQ measures were used as dependent variables, and age, AS-Total score, HADS-A, HADS-D, MOS-SF-PCS, MOS-SF-MCS and LOT-Optimism as independent variables, using the backward exclusion selection procedure. As can be seen in Table 4, optimism and physical QoL emerged as significant independent predictors of PE-Total score, whereas optimism, AS measure and depression symptoms were significant independent predictors of the PH-Total score.

**Table 4:** Standardized regression coefficients, *t* and *p* values for the variables of interest regressed on expectations and hopes measures

Independent variables	Dependent variables																				
	Age			AS-Total score			HADS-A			HADS-D			MOS-SF-PCS			MOS-SF-MCS			LOT-Optimism		
	β	t	*p*	β	t	*p*	β	t	*p*	β	t	*p*	β	t	*p*	β	t	*p*	β	t	*p*
PH-Total score	**.22**	**2.08**	**.04**	**− .47**	**− 3.66**	**.00**	.13	1.09	.28	**− .24**	**− 2.39**	**.02**	− .11	− .85	.40	− .14	− 1.16	.26	**.48**	**5.20**	**.00**
PH-Abstract	**.31**	**3.57**	**.00**	**− .59**	**− 6.62**	**.00**	.07	.68	.50	**− .21**	**− 2.55**	**.02**	− .09	− .85	.40	− 06	− .62	.54	**.43**	**5.65**	**.00**
PH-Concrete	**− .30**	**− 2.23**	**.03**	− .00	− .02	.98	.24	1.48	.15	− .12	− .78	.44	.02	.12	.90	**− .33**	**− 2.03**	**.04**	.26	1.91	.06
PE-Total score	.07	.52	.61	− .26	− 1.76	.087	.15	1.15	.26	.05	.38	.70	**− .41**	**− 3.26**	**.00**	.09	.62	.54	**.37**	**3.47**	**.00**
PE-Abstract	.10	.87	.39	− 2.71	− 1.98	.054	.13	1.07	.29	.02	.21	.83	**− .48**	**− 4.13**	**.00**	.09	.74	.46	**.36**	**3.61**	**.00**
PE-Concrete	.10	.68	.49	− .11	− .72	.82	.16	− .72	.47	.16	1.04	.30	.01	.08	.93	− .10	.68	.49	.16	1.06	.30

Bold values indicate predictors significant at *p* < .05

*PE* preoperative expectations, *PH* preoperative hopes

Specific relationships between expectations and hope and the other variables of interest were also examined, by taking the level of abstraction of life domains into account. In this prospect, four additional regression analyses have been performed, with PE-Abstract, PH-Abstract, PE-Concrete and PH-Concrete as dependent variables, and age, HADS-A, HADS-D, MOS-SF-PCS-, MOS-SF-MCS, LOT-Optimism and AS-Total score as independent variables, using the backward exclusion selection procedure. As can be seen in Table 4, age, actual state, optimism and depression symptoms emerged as significant independent predictors of PH-Abstract, whereas optimism and physical QoL were significant independent predictors of the PE-Abstract. Age and mental QoL emerged as significant independent predictors of the PH-Concrete, whereas none of the variables predicted the PE-Concrete.

### Group comparisons

**Fig.1:**
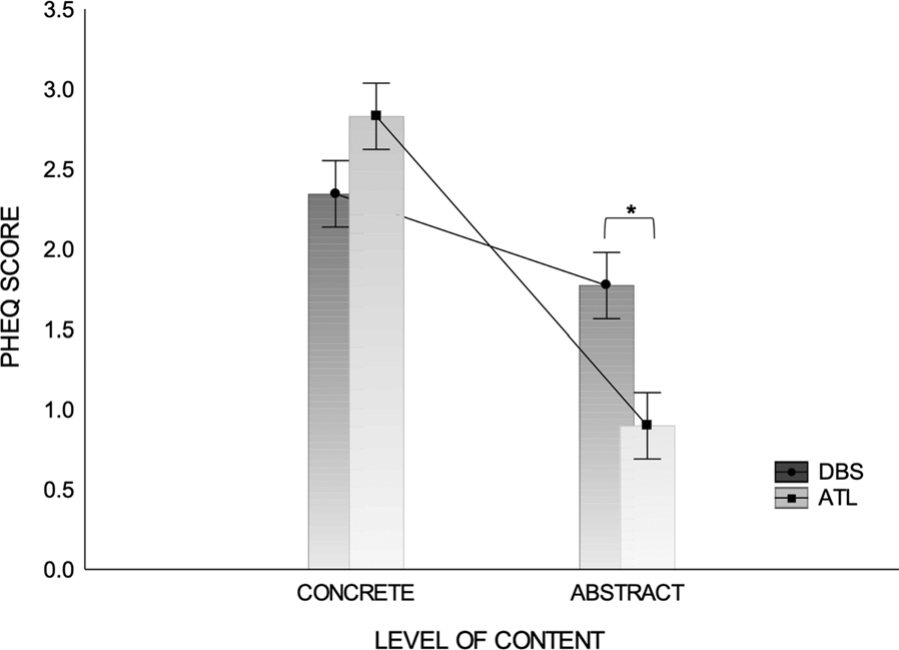
Interaction between Group and Level of content. *Significant mean differences

Future oriented cognitions across the two groups of patients were explored by means of a 2 (Type of content: Hope, Expectations) × 2 (Level of content: Concrete, Abstract) × 2 (Type of neurosurgery: DBS vs. ATL) factorial ANOVA. A main effect of type of content was observed suggesting that candidates for neurosurgery expressed higher desire of changes than realistic expectations regarding the outcome of surgery *F*(1, 192) = 21.59, *p* <.000, *η*^*2*^ = .10 (a medium effect size, according to Cohen’s criteria; [60]). The main effect of level of content was significant, suggesting that patients expressed expectations and hope predominantly regarding concrete aspects of QoL, *F*(1, 192) = 141.91, *p* < .000, *η*^*2*^ = .42 (a large effect size, according to Cohen’s criteria). There was no main effect of group nor interaction Group × Type of content. Results revealed an interaction Group x Level of content *F*(1, 192) = 41.77, *p* < .000, *η*^*2*^ = .18 (a large effect size, according to Cohen’s criteria). Bonferroni post hoc tests suggest the two groups had comparable levels of concrete representations but DBS candidates had significantly higher abstract representations as compared to ATL patients (*p* < .001) (see Figure 1). Finally, there was no triple interaction Type of content x Level of content x Group.

## Discussion

The aim of this study was to develop a tool assessing future-oriented cognitions in the context of functional neurosurgery. The measure is novel in combining two types of preoperative representations, hope and realistic expectations. The results suggested that the PHEQ is a reliable instrument with satisfying psychometric properties. Previous findings regarding the relationships between preoperative representations and dispositional optimism [53] have been replicated in the present study, supporting the idea that expectations, hope and optimism convey a general construct that can be conceptualized as an anticipatory state and beliefs about the future [61, 62].

The pattern of correlations observed in this study further support the idea that hope and expectations are two distinct, although linked constructs [45]. More specifically, expectations were highly correlated with hope, but these two constructs showed distinct patterns of associations with other measures. Lower preoperative expectations were associated with low optimism and high physical QoL, while low preoperative hope was associated with high actual state, low optimism, and high depression symptoms, supporting the idea that patients exhibiting depressive attitudes tend to demonstrate hopelessness [38]. Consistent with previous studies, the perceived actual state regarding various life domains appeared to be strongly related to the desire for positive outcomes following surgery. However, while in some studies the actual state assessment and preoperative representations were predominantly connected to illness-related issues rather than cognitive, psychological and social problems [40], our results showed that lower subjective evaluation of the actual state is associated with an increased desire for postoperative improvements in psychological and social domains.

At a group level, patients reported hope for improvements that was significantly higher than realistic expectations. This suggests that they may experience strong desires for substantial changes following neurosurgery that may, at the same time, be perceived as poorly probable. Discrepancies between desire of outcomes and evaluation of the probability that such outcomes may occur might interfere with postoperative adjustments process. This hypothesis should be directly explored in a longitudinal study aimed at exploring the way expectations and hope as measured by the PHEQ may predict, at least partly, the frequently reported BoN syndrome following surgery.

In order to assess the clinical value of the new questionnaire, the PHEQ was administered to candidates for DBS and ATL procedures. First, our results indicated that patients did not expect a complete recovery following treatment, which is consistent with other studies [43]. Our results also showed that candidates for neurosurgery had preoperative representations of outcomes that were generally more attuned towards concrete surgery outcomes. This result is in line with Wilson et al. [36] who found that seizure cessation is the most frequently endorsed expectation in candidates for ATL. On the other hand, Baca et al. [39] and Wheelock et al. [35] suggested that patients may feel that symptoms reduction is implicit to other postoperative changes, and therefore, they do not formerly endorse it as a discrete expectation, which would explain the fact that symptom reduction was endorsed by less than half of the participants in Baca et al. [39].

In this study we also explored the possibility that candidates for DBS and ATL conceptualized preoperative representations differently. Our results suggested that patients with PD had significantly higher abstract representations (*to feel good about myself, to feel more like myself*) as compared to ATL patients. This result is somewhat surprising considering previous findings suggesting that candidates for ATL are more prone to endorse expectations related to psychological and social domains (e.g., improved personality or social circle, see Wilson et al. [34]). Our findings suggest that candidates for DBS are particularly at risk of having unspecific, psychological, or interpersonal preoperative representations, which may lead to dissatisfaction with the overall outcome despite significant improvements in objective measures [3, 63].

Before concluding, some important limitations of the present study should be emphasized. First, the nature of the relationships found between the PHEQ and the other related constructs should be further refined, as the potential confounding effect of other factors, such as cognition, disease severity or duration were not controlled for, although patients with severe cognitive deficits were excluded during selection for DBS or ATL (based on a cutoff score of 130 on the Mattis Dementia Rating Scale). It is noteworthy that an important factor that potentially affects presurgical expectations has not been explicitly controlled in this study, namely the attitude of practitioners in providing information related to surgery. For instance, the extent to which a neurologist delivers an optimistic perspective or highlights predominantly potential benefits vs. a realistic perspective focused on risks and adverse effects, may affect the way candidates will perceive the outcomes. It should be noted however that in our study information was given to the candidates by means of a standardized brochure which fully explained all surgery aspects and by the neurologist’s explanations that were putatively comparable from one candidate to another. Further studies as well as health care providers should take the aforementioned parameter into account. Additionally, considering the small size of our sample, future studies should re-examine the complex factor structure of the PHEQ in a bigger sample by means of a confirmatory factor analysis. Finally, other studies should directly examine the relations between the PHEQ dimensions and existing constructs assessing treatment expectations, especially in the case of debilitating neurological diseases.

## Conclusions

By and large, this research has important implications for the clinical management of candidates for functional neurosurgery. A better characterization of particular features of preoperative expectations may help clinicians to better understand what is important for their patients and enhance their adherence to treatment. Moreover, measuring changes in or fulfillment of expectations and their impact on satisfaction and clinical outcomes may help clinicians to optimize treatment strategies. Importantly, implementing tailored preoperative preparation consisting of cognitive restructuration of unsuitable expectations may prevent adverse events, thereby improving postoperative psychosocial adjustment and QoL.

## Supplementary Information

**Additional file 1: Table A1.** Descriptive statistics (Mean, Standard Deviation, lowest and highest scores) for the two future oriented PHEQ dimensions. Note: *PH* Preoperative hope scale, *PE* Preoperative expectations scale.

## Data Availability

The de-identified data that support the findings of this study are available on the Figshare repository https://doi.org/10.6084/m9.figshare.14522778.v4.

## Abbreviations

AS: Actual State
ATL: Anterior temporal lobectomy
BoN: Burden of normality
DBS: Deep brain stimulation
HADS: Hospital Anxiety and Depression Scale
KMO: Kaiser–Meyer–Olkin
LOT: Life orientation test
MCS: Mental component summary score
MOS-SF-36: Medical outcome study short form
PCS: Physical component summary score
PD: Parkinson's disease
PE: Preoperative expectations
PH: Preoperative hope
PHEQ: The Preoperative Hope and Expectation Questionnaire
QoL: Quality of life
UPDRS: Unified Parkinson’s Disease Rating Scale III

## Footnotes

1 Behaviors associated with being sick such as domestic, social, recreational, vocational underactivity or focusing on novel somatic or cognitive complaints.
