# Supplementary material for: Assessing preoperative hope and expectations related to functional neurosurgery: a new questionnaire
Source: BMC Psychol. 2022 Mar 4;10:53. doi: 10.1186/s40359-022-00766-z (PMC8897841; doi:10.1186/s40359-022-00766-z)
Supplement: Supplementary file 1 — Additional file 1: Table A1. Descriptive statistics (Mean, Standard Deviation, lowest and highest scores) for the two future oriented PHEQ dimensions. Note: PH Preoperative hope scale, PE Preoperative expectations scale. [file 40359_2022_766_MOESM1_ESM.docx]

|  |  | PH | | | | |  | PE | | | | |
| --- | --- | --- | --- | --- | --- | --- | --- | --- | --- | --- | --- | --- |
| *#* | *Item* | *Valid n* | *Mean* | *SD* | *Min* | *Max* |  | *Valid n* | *Mean* | *SD* | *Min* | *Max* |
| 1 | To be satisfied with my life | 50 | 2.96 | 1.03 | 0.0 | 4.0 |  | 50 | 2.10 | 1.13 | 0.0 | 4.0 |
| 2 | To reduce symptoms of my disease | 50 | 3.50 | 0.58 | 2.0 | 4.0 |  | 50 | 2.96 | 0.57 | 1.0 | 4.0 |
| 3 | To be independent in my personal care | 50 | 0.92 | 1.43 | 0.0 | 4.0 |  | 50 | 0.80 | 1.19 | 0.0 | 4.0 |
| 4 | To feel good about myself | 50 | 1.26 | 1.35 | 0.0 | 4.0 |  | 50 | 1.10 | 1.22 | 0.0 | 3.0 |
| 7 | To be satisfied with my physical appearance | 50 | 1.06 | 1.18 | 0.0 | 4.0 |  | 50 | 0.72 | 0.99 | 0.0 | 3.0 |
| 9 | To be satisfied with my social life (family, friends) | 50 | 1.42 | 1.46 | 0.0 | 4.0 |  | 50 | 0.98 | 1.15 | 0.0 | 4.0 |
| 10 | To be able to achieve my projects | 50 | 2.06 | 1.43 | 0.0 | 4.0 |  | 50 | 1.56 | 1.18 | 0.0 | 4.0 |
| 11 | To be able to participate in leisure activities (e.g., sports, travel) | 50 | 1.98 | 1.41 | 0.0 | 4.0 |  | 50 | 1.58 | 1.25 | 0.0 | 4.0 |
| 12 | To feel more like myself | 50 | 1.14 | 1.43 | 0.0 | 4.0 |  | 50 | 0.74 | 1.14 | 0.0 | 4.0 |
| 13 | To be satisfied with my intellectual functioning (e.g., concentration, memory) | 50 | 1.94 | 1.39 | 0.0 | 4.0 |  | 50 | 1.46 | 1.23 | 0.0 | 4.0 |
| 17 | Not to experience negative feelings (e.g., sad, anxious) | 50 | 1.48 | 1.47 | 0.0 | 4.0 |  | 50 | 1.16 | 1.23 | 0.0 | 4.0 |
| 18 | To feel comfortable in social situations (e.g., outings, parties) | 50 | 2.08 | 1.32 | 0.0 | 4.0 |  | 50 | 1.80 | 1.28 | 0.0 | 4.0 |
| 21 | To reduce physical pain | 50 | 1.56 | 1.54 | 0.0 | 4.0 |  | 50 | 1.30 | 1.42 | 0.0 | 4.0 |
| 22 | To get off medications | 50 | 3.26 | 1.10 | 0.0 | 4.0 |  | 50 | 2.520 | 1.129 | 0.0 | 4.0 |
